# Supplementary material for: Systematic Design of Polypyrrole/Carbon Fiber Electrodes for Efficient Flexible Fiber-Type Solid-State Supercapacitors
Source: Nanomaterials (Basel). 2020 Jan 30;10(2):248. doi: 10.3390/nano10020248 (PMC7075248; doi:10.3390/nano10020248)
Supplement: Supplementary file 1 [file nanomaterials-10-00248-s001.pdf]

Supplementary Materials:

Systematic Design of Polypyrrole/Carbon Fiber Electrodes for Efficient Flexible Fiber-type Solid-state Supercapacitors

Yu-Shun Sung [1,†] and Lu-Yin Lin [1,2,†,*]

[1] Department of Chemical Engineering and Biotechnology, National Taipei University of Technology, Taipei 10608, Taiwan

[2] Research Center of Energy Conservation for New Generation of Residential, Commercial, and Industrial Sectors, Taipei 10608, Taiwan

[†] These authors contributed equally to this work.

[*] Correspondence: lylin@ntut.edu.tw; Tel: +886–2–2771–2171 ext. 2535; Fax: +886–2–2731–7117

Table S1. The C_L values for the PPy@CF electrodes prepared using 1 mA and 10 min for electropolymerization in different electrolytes.

Electrolyte	C_L (mF/cm)
0.0 M PPy	0.44
0.5 M PPy	4.83
1.0 M PPy	6.69
0.5 M PPy + $NaClO_4$	78.54
1.0 M PPy + $NaClO_4$	93.75
1.5 M PPy + $NaClO_4$	85.08

Table 2. The specific capacitances and charge-transfer resistances for the PPy@CF electrodes prepared using 10 min at different applied currents for electropolymerization in the electrolyte containing 0.5 M pyrrole and 0.3 M $NaClO_4$.

Current (mA)	C_L (mF/cm)	C_M (F/g)	R_S (Ω)	R_{ct} (Ω)
10	126.8	211.4	3.32	1.68
20	211.9	238.1	2.20	0.15
30	229.0	200.9	3.00	0.35
40	244.9	178.8	2.13	0.16
50	180.9	110.3	3.40	0.60

Table S3. The specific capacitances calculated using the CV curve at 5 mV/s and charge-transfer resistances for the PPy@CF electrodes prepared using different times at 40 mA for electropolymerization in the electrolyte containing 0.5 M pyrrole and 0.3 M $NaClO_4$. The loading mass of the active material on the substrate was also listed in this table.

Time (min)	C_L (mF/cm)	C_M (F/g)	R_S (Ω)	R_{ct} (Ω)	Loading mass (mg)
10	394.3	277.7	3.13	0.883	14.2
20	921.5	308.2	2.37	0.345	29.9
30	1140.0	287.2	2.55	0.386	39.7

Table S4. The specific capacitances for the PPy@CF electrodes prepared using different times for electropolymerization measured using CV curves at different scan rates.

Time (min)	Scan rate (mV/s)	C_L (mF/cm)	C_F (F/g)
10	1	467.4	329.1
	2	452.7	318.8
	5	394.3	277.7
	10	370.9	261.2
	15	350.2	246.6
	20	327.5	230.6
20	1	970.9	324.7
	2	966.4	323.2
	5	921.5	308.2
	10	756.3	252.9
	15	579	193.6
	20	444	148.5
30	1	1352	340.6
	2	1270	319.8
	5	1140	287.2
	10	825.5	207.9
	15	577.3	145.4
	20	416.2	104.8

Table S5. The specific capacitances for the PPy@CF electrodes prepared using different times for electropolymerization prepared using the GC/D curves at different current densities.

Time (min)	Current density (A/g)	C_L (mF/cm)	C_F (F/g)
10	0.5	1352.3	340.6
	1	1269.5	319.8
	1.5	1140.3	287.2
	2	825.5	207.9
	3	577.3	145.4
20	0.5	207.4	146.1
	1	169.4	119.3
	1.5	161.3	113.6
	2	150.1	105.7
	3	149.1	105.0
30	0.5	575.6	192.5
	1	425.0	142.1
	1.5	394.0	131.8
	2	358.8	120.0
	3	294.7	98.6

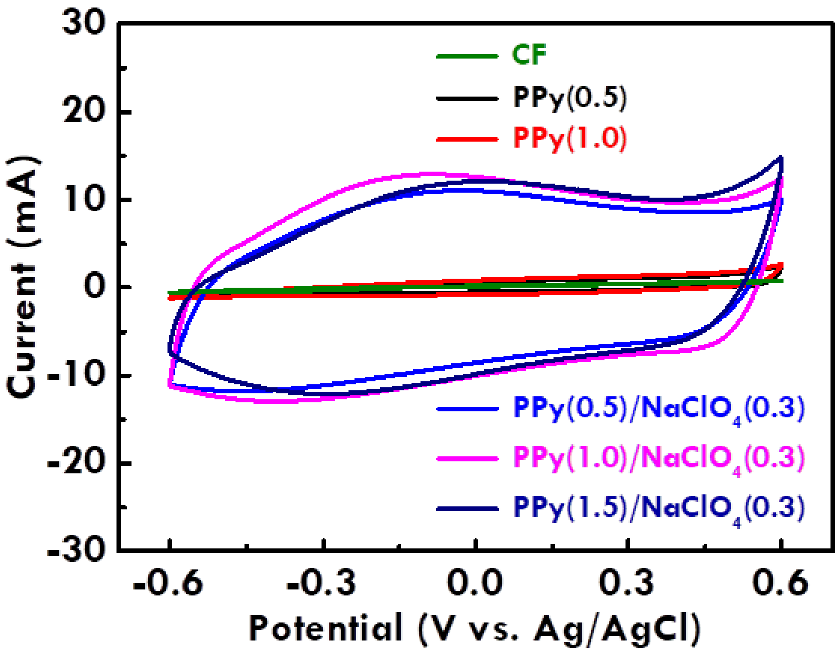

Figure S1. The CV curves for the PPy@CF electrodes prepared using 1 mA and 10 min for electropolymerization in different electrolytes.

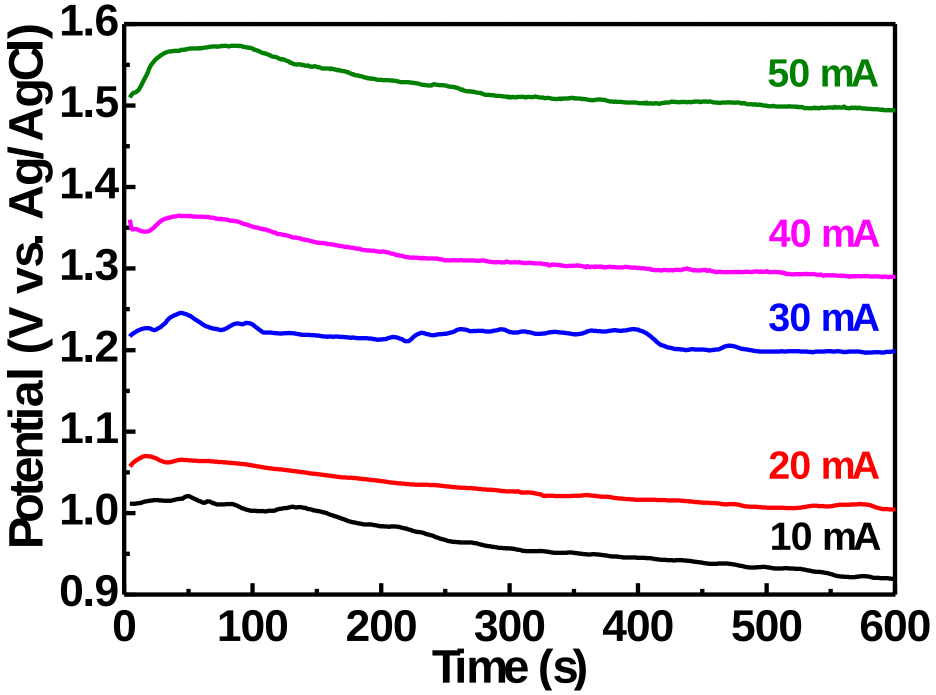

Figure S2. Chronopotentiometric curves for the electrodes prepared using 10, 20, 30, 40 and 50 mA.

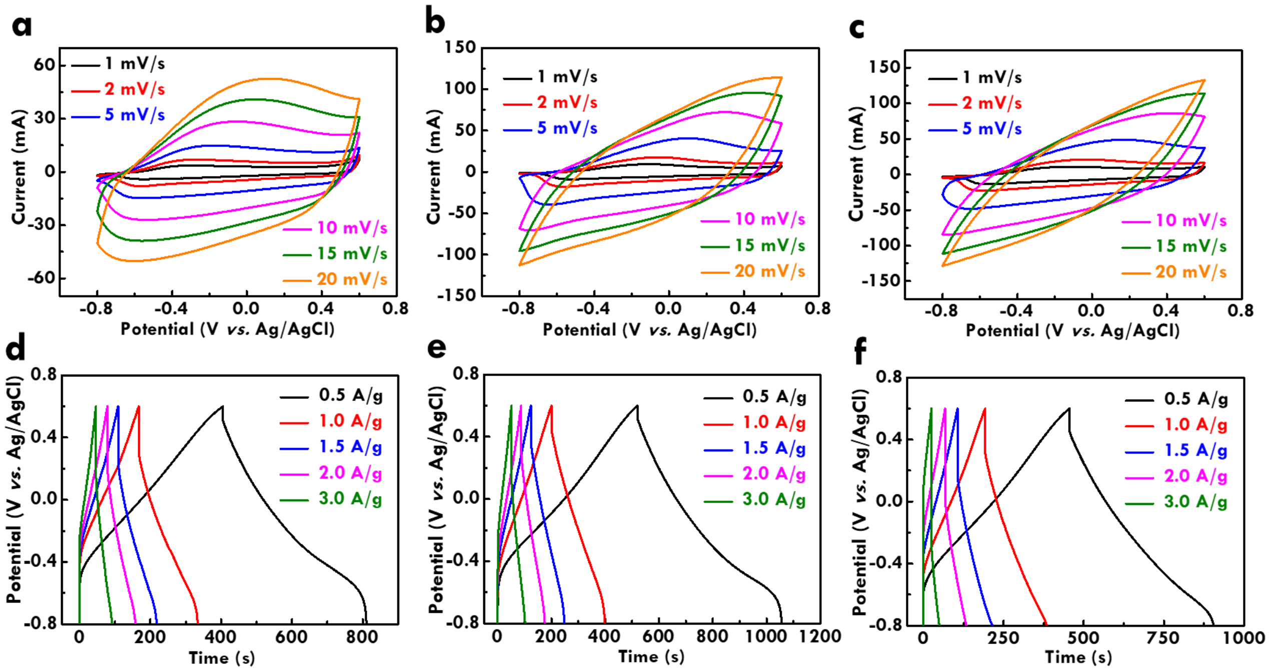

Figure S3. The CV curves at different scan rates for PPy@CF prepared using (a) 10, (b) 20, and (c) 30 min; GC/D curves at different current densities for PPy@CF prepared using (d) 10, (e) 20, and (f) 30 min and the applied current of 40 mA in the electrolyte containing 0.5 M pyrrole and 0.3 M $NaClO_4$.

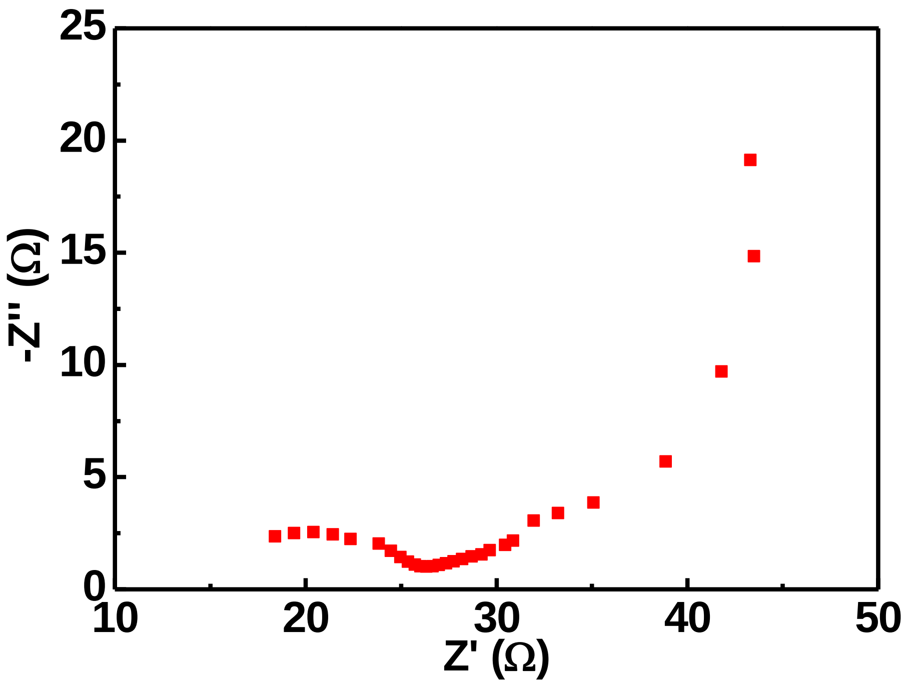

Figure S4. The Nyquist plot for the FSC with H_3PO_4/PVA electrolyte.
